# Residual Mechanical Properties and Constitutive Model of High-Strength Seismic Steel Bars through Different Cooling Rates

**DOI:** 10.3390/ma14020469

**Published:** 2021-01-19

**Authors:** Xianhua Yao, Peiqiao Qin, Junfeng Guan, Lielie Li, Min Zhang, Yongwei Gao

**Affiliations:** 1 School of Civil Engineering and Communication, North China University of Water Resources and Electric Power, Zhengzhou 450045, China; yaoxianhua@ncwu.edu.cn (X.Y.); qinpeiqiao@163.com (P.Q.); 1000-lilili@163.com (L.L.); gaoyongwei@ncwu.edu.cn (Y.G.); 2 School of Architectural Engineering, Zhengzhou University of Industrial Technology, Zhengzhou 451150, China

**Keywords:** 600 MPa seismic steel bars, high temperature, cooling modes, mechanical properties, constitutive models

## Abstract

In this study, the high-temperature test (i.e., temperature to 1000 °C) is conducted on 600 MPa seismic steel bars, and its residual mechanical properties and constitutive relations are investigated though three cooling rates, i.e., under air, furnace, and water-cooling conditions. Results show that three cooling methods have significant effects on the apparent characteristics of 600 MPa steel bars, when the heating temperature is greater than 600 °C. In addition, the ultimate and yield strength of steel bars have been significantly affected by different cooling methods, with increasing heating temperature. However, the elastic modulus is significantly not affected by temperature. Furthermore, the elongation rate after fracture and the total elongation rate at the maximum force do not change significantly, when the heating temperature is less than 650 °C. The elongation rate, after fracture, and the total elongation rate, at the maximum force, have different changes for three cooling methods. The degeneration of the stress–strain curves occurs when the heating temperature is high. The two-fold line, three-fold line, and Ramberg–Osgood models are developed based on the stress–strain curve characteristics of steel bars after cooling. The fire resistance of 600 MPa steel bars of reinforced concrete structure is analyzed, which provides a basis for post-disaster damage assessment, repair, and reinforcement of the building structure.

## 1. Introduction

In recent years, high-strength steel bar has gradually become the focus of intensifying research and discussion. High-strength steel bars have been widely used in various engineering fields. For example, the use of high-strength steel bars in high-strength concrete walls and columns, as well as in railway crossings, can effectively reduce the amount of steel bar used, save costs, and reduce steel bar congestion within structures [1,2,3,4]. The probability of fire occurrence is relatively high and extremely destructive, especially for high-rise buildings, super high-rise buildings, and deep underground structures, which will cause casualties and inestimable economic losses [5]. As the internal temperature of the reinforced concrete structure increases, the performance of the steel bar significantly deteriorates, which eventually leads to decreasing bearing capacity of the building structure [6,7,8]. Some developed countries have halted the use of lower-strength steel bar.

Globally, the development of steel bars shows a continuous improvement in production technology, strength, ductility, and service life. At present, many countries have developed and applied steel bars with high strength, high-temperature resistance, corrosion resistance, and other comprehensive properties. According to ACI 318-14 [9] and EN1992-1-1:2004 [10], the maximum yield strength of ordinary stressed steel bars can reach 550–600 MPa. The grades of ribbed steel bars listed in the international standard ISO6935-2 are 300 MPa, 400 MPa, and 500 MPa [11]. In the United States, United Kingdom, Japan, and other countries, steel bars with a strength grade greater than 400 MPa are generally used. In France, Germany, Australia, and other countries, steel bars with a strength grade of 500 MPa are used; for a higher strength of 600 MPa, steel bar application has been greatly improved.

Outinen et al. [12] used the transient-state and steady-state tensile test method to study the mechanical properties of structural steel under the effect of increased temperature, such as yield strength, elastic modulus, and thermal elongation. Heidarpour et al. [13] studied the mechanical properties of high-strength steel at high temperature and put forward the equations for predicting yield strength and ultimate strength reduction factors for structural steel from room temperature to 600 °C—the results of which could be used for reference in the design of fire protection engineering structures. Chiew et al. [14] investigated the mechanical properties of S690 grade high-strength steel (HSS) after high-temperature reheating quenching and tempering (RQT), and the residual strength after heating and cooling was also studied. RQT-S690 HSS had good heating performance below 400 °C, but its strength deteriorates significantly at higher temperatures. Chen et al. [15] studied the mechanical properties of high-strength steel and mild structural steel at high temperature by conducting a series of transient-state and steady-state experiments. The decreasing trend of yield strength and elastic modulus of high-strength steel and mild steel were similar for temperatures ranging from 22 °C to 540 °C. Kodur et al. [16] studied the constitutive model of steel under high temperature under the current American and European standards. High temperatures significantly affect the creep of steel bars and decrease the fire resistance of structures. Kumar et al. [17] simulated the effect of earthquake damage on the mechanical properties of steel reinforcement at high temperature. Results showed that the heating effect is significant only when the heating temperature is greater than or equal to 400 °C. The results could be used to predict the residual bearing capacity of reinforced concrete structures subjected to fire and earthquakes. Elghazouli et al. [18] carried out room-temperature and high-temperature tests on hot-rolled and cold-worked steel bars in order to study the mechanical properties of steel bars after high-temperature action, which was critical for the reliable assessment of structural component performance changes in response to fire and post-fire repair. Bompa et al. [19] studied the effect of high temperature on the mechanical properties of hot-rolled steel bars with internal threaded connectors and analyzed the test results using DIC (digital image correlation) technology. The test results could be used to evaluate the current design criteria for steel and mechanical joints at high temperature.

In order to reduce the amount of energy and steel bar used in construction, GB/T1499.2-2018 added 600 MPa heat-tied ribbed steel bars in concrete steel, among which, the lower yield strength of HRB600 steel bars is *R*_*eL*_ ≥ 600 MPa, the tensile strength *R*_*m*_ ≥ 730 MPa, the elongation after fracture rate *A* ≥ 14%, and the maximum total elongation rate *A*_*gt*_ ≥ 7.5% [20]. Sun et al. [21] investigated the changes in the mechanical characteristics of 600 MPa steel bars at high temperature. Results showed that the yield strength, ultimate strength, and elastic modulus of steel bars cooled in different ways gradually decreased after 625 °C. Concrete structures with seismic steel bar can properly control the stiffness of building structures in order to provide greater ductility during an earthquake, which can consume earthquake energy and improve the safety of the building [22]. Guan et al. [23] studied the mechanical properties and constitutive relationship of 600 MPa seismic reinforcement at indoor temperature using tensile tests and obtained the full stress–strain curves. Results showed that the mechanical properties of the seismic steel bars were good, and all the indexes met the requirements of domestic and foreign codes. Simultaneously, two-fold and three-fold line constitutive models with 600 MPa seismic reinforcement were proposed.

Hot-rolled steel bar is a kind of steel bar formed by hot rolling and natural cooling. Hot-rolled steel bar has a certain strength but also has good plasticity, toughness, weldability and bonding strength between the steel bar and concrete. This kind of reinforcement has the advantages of high strength, low material consumption, good anchorage, and stable prestress. Current scholars primarily focus on the mechanical properties of 400 MPa, 500 MPa, and 600 MPa hot-rolled steel bars, however, there is no relevant examination of the high-temperature properties of 600 MPa heat-treated high-strength seismic steel bars. In addition, there is still a gap in the constitutive model comparative study of 600 MPa heat-treated high-strength seismic steel bar after high-temperature cooling. The studies of the three cooling methods after high temperature effects the 600 MPa seismic reinforcement are also quite scarce. In addition, different heating temperatures have different effects, the current research on heating temperature scope are also less.

In this paper, the static tensile properties of 600 MPa heat-treated high-strength seismic steel bar after high-temperature cooling are systematically studied by conducting the heating temperature gradient of the target and adopting different cooling methods. The variation in steel bar mechanical properties with changing temperature for different cooling modes is obtained. In addition, according to the stress–strain curve characteristics of 600 MPa heat-treated high-strength seismic steel bar after high-temperature cooling, a variety of reasonable constitutive models are proposed and compared. This study not only accurately evaluates the fire resistance of 600 MPa seismic-reinforced concrete structures but also provides a basis for post-disaster damage assessment, repair and reinforcement of building structures.

## 2. Materials and Methods

### 2.1. Materials

In this test, 600 MPa high-strength seismic steel bars with a diameter of 14mm and 18mm, which were specially heat treated, were analyzed. The yield strength measured values (*R*_*eL*_) of steel bars range from 636 MPa to 662 MPa, the ultimate strength measured values (*R*_*m*_) range from 826 MPa to 866 MPa, the strength yield ratio ($R_{m}^{0}$/ $R_{eL}^{0}$) ranges from 1.28 to 1.31, the elongation rate after fracture values (*A*) were all greater than 14%, and the total elongation rate at maximum force values (*A*_*gt*_) were all greater than 9%. All mechanical indexes for the selected steel bars met the requirements of code GB50010-2010 [24], GB/T1499.2-2018 [20], American standard ASTM A706/A706M-14 [25], and European standard EN1992-1-1:2004 [10]. In addition, the shape and size of the steel bars met the requirements of ribbed steel bars in GB/T1499.2-2018 [20] and GB/T228.1-2010 [26].

### 2.2. Methods

**Figure 1:**
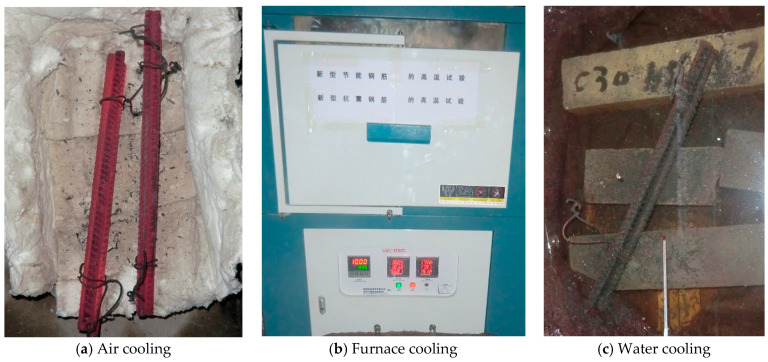
Three ways of cooling.

**Figure 2:**
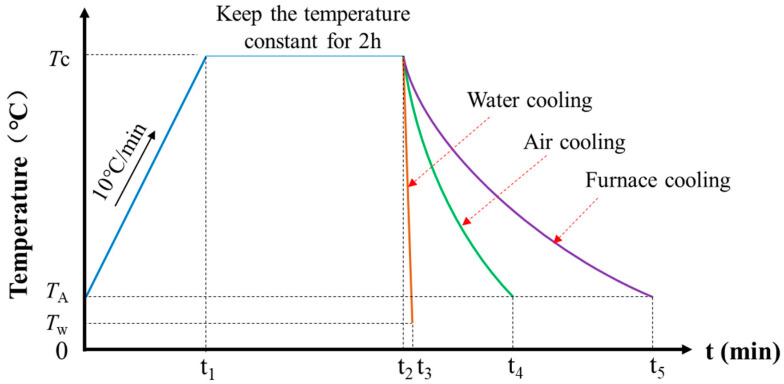
Schematic diagram of heating temperature and cooling system. (*T*c denotes target temperature; *T*_A_ is ambient temperature; *T*_w_ represents water temperature).

The high-temperature test used a box-type electric furnace made by Luoyang Juxing Kiln Co., Ltd. (Luoyang, China), which can be heated up to 1200 °C. The test included 15 temperature gradients of 100, 200, 300, 400, 500, 550, 600, 650, 700, 750, 800, 850, 900, 950, and 1000 °C, with room temperature 20 °C as the control group. Each temperature state contained three steel bar samples. After the high-temperature test was completed, the steel bars were cooled by air cooling, furnace cooling, and water cooling (Figure 1). The heating temperature and cooling system can be seen in Figure 2. After cooling, the changes in the apparent characteristics of the steel bar specimens were observed.

**Figure 3:**
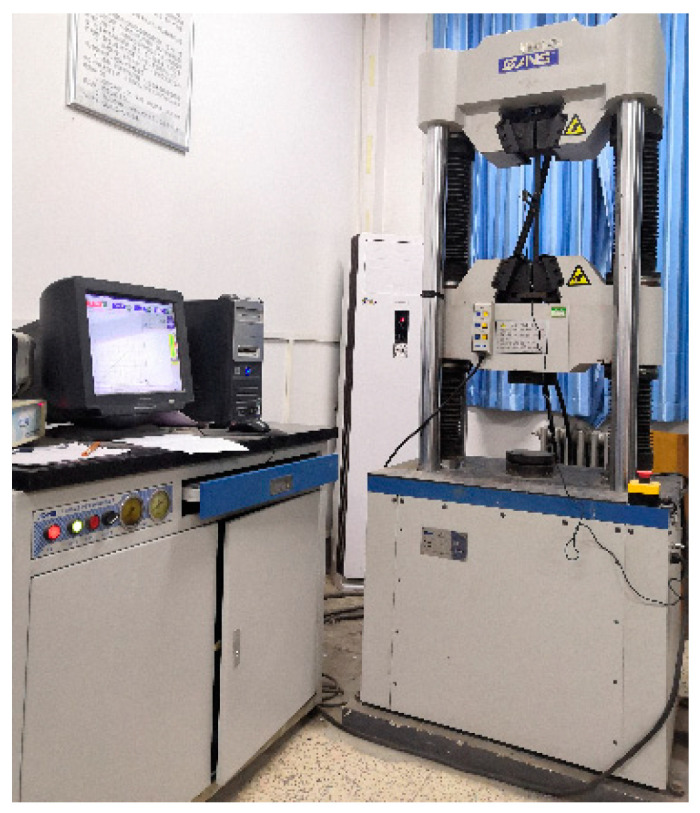
Tensile test.

**Figure 4:**
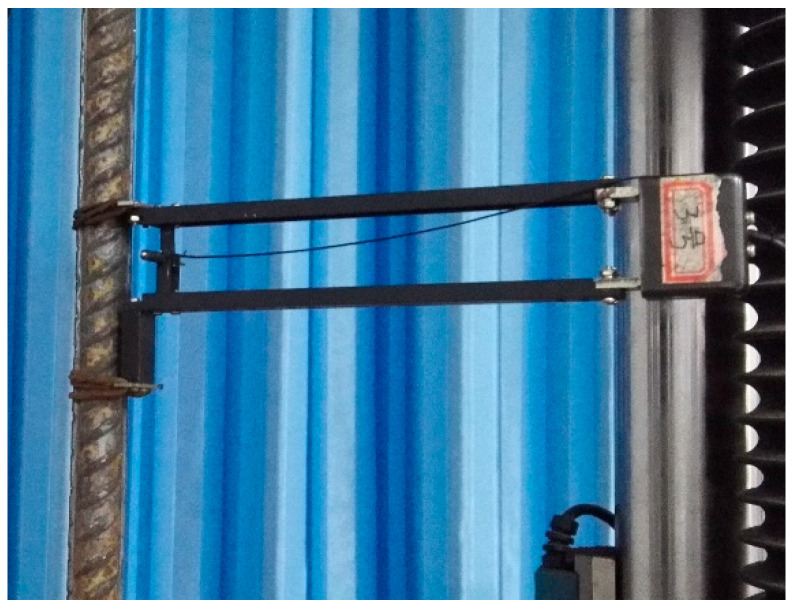
High-precision extensometer.

A SHT4605 hydraulic servo universal testing machine (Shanghai New Sansi Measuring Instrument Manufacturing Co., Ltd., Zhengzhou, China) was used to conduct direct tensile tests on steel bars after high-temperature cooling (Figure 3). The tensile test of steel bars was conducted according to the GB/T228.1-2010 tensile test for metal materials part 1: test method at room temperature [26]. In the test, a 10 MPa/s force control loading mode was adopted, and the steel bar deformation was measured throughout the whole process using a YSJ50-25 high-precision extensometer (gauge length 50 mm, maximum measured deformation 25 mm), allowing the measured load—deformation curves of seismic steel bars could be obtained (Figure 4) [23].

## 3. Results and Discussion

### 3.1. Morphology Analysis

**Figure 5:**
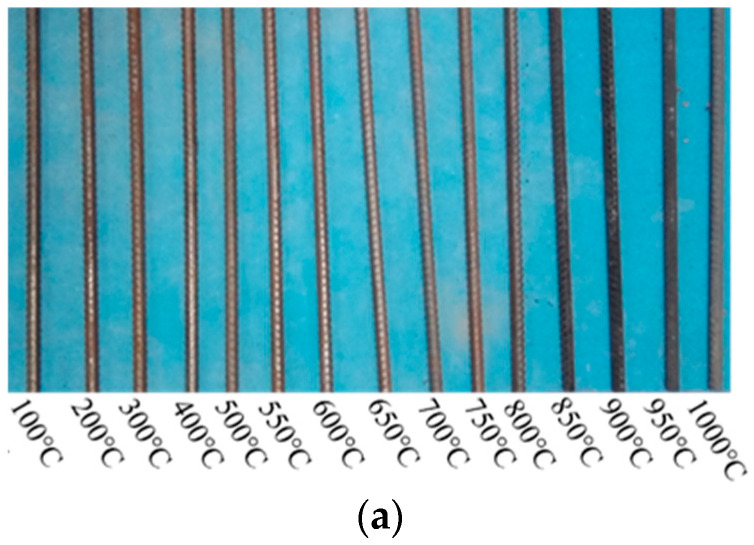

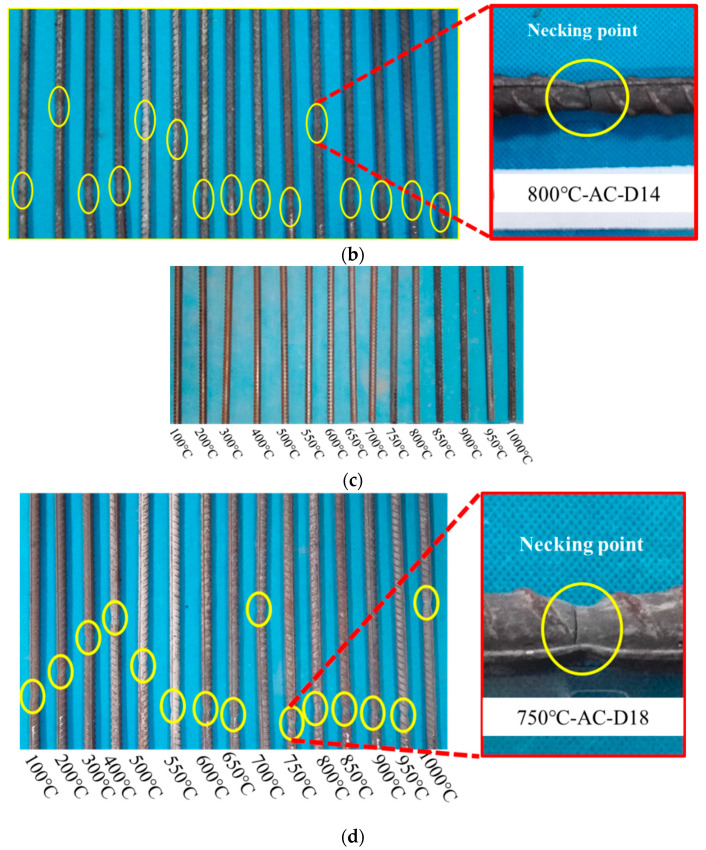
Appearance of steel bar after air cooling. (**a**) The surface color of 14 mm diameter steel bars changing with temperature after high-temperature cooling; (**b**) the tensile fracture sections of 14 mm diameter steel bars under air cooling, both show significant necking and fractures in the shape of a silver cup cone; (**c**) the surface color of 18 mm diameter steel bars changing with temperature after high-temperature cooling; (**d**) the tensile fracture sections of 18 mm diameter steel bars under air cooling, both show significant necking and fractures in the shape of a silver cup cone.

**Figure 6:**
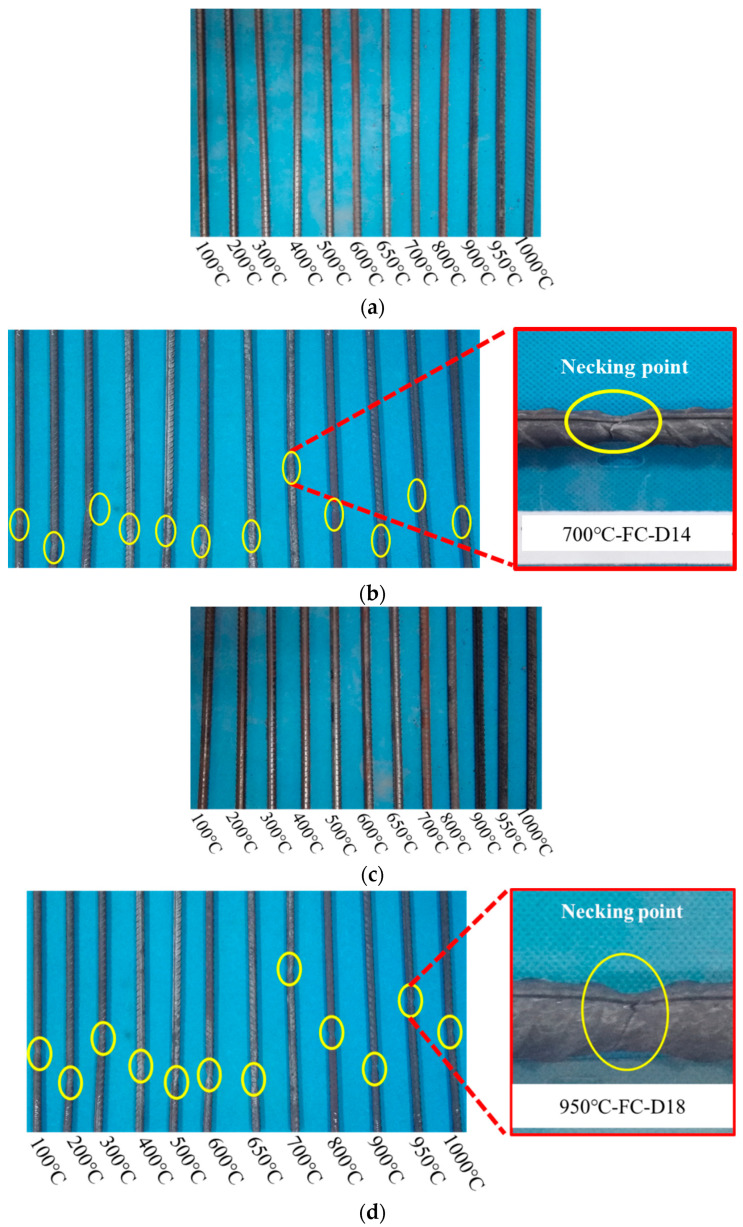
Appearance of steel bar after furnace cooling. (**a**) The surface color of 14 mm diameter steel bars changing with temperature after high-temperature furnace cooling; (**b**) the tensile fracture sections of 14 mm diameter steel bars under furnace cooling, both show significant necking and fractures in the shape of a silver cup cone; (**c**) the surface color of 18 mm diameter steel bars changing with temperature after high-temperature furnace cooling; (**d**) the tensile fracture sections of 18 mm diameter steel bars under furnace cooling, both show significant necking and fractures in the shape of a silver cup cone.

**Figure 7:**
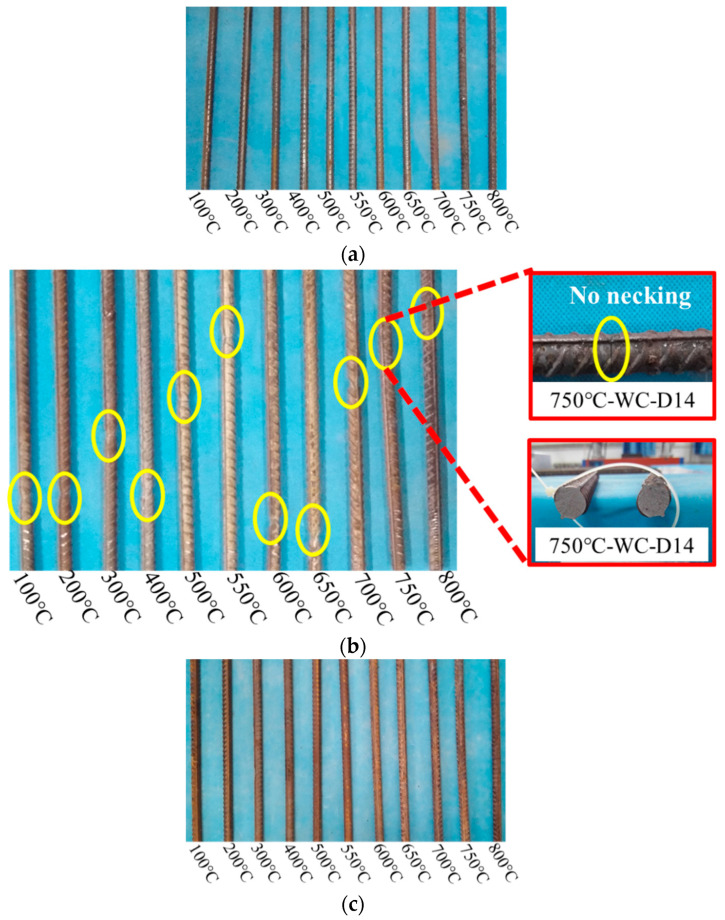

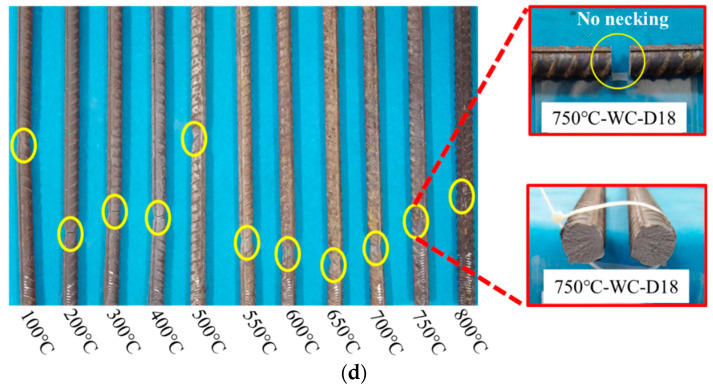
Appearance of steel bar after water cooling. (**a**) The surface color of 14 mm diameter steel bars changing with temperature after high-temperature water cooling; (**b**) the tensile fracture sections of 14 mm diameter steel bars under water cooling, both show significant necking and fractures in the shape of a silver cup cone; (**c**) the surface color of 18 mm diameter steel bars changing with temperature after high-temperature water cooling; (**d**) the tensile fracture sections of 18 mm diameter steel bars under water cooling, both show significant necking and fractures in the shape of a silver cup cone.

Figure 5, Figure 6 and Figure 7 show the surface color of 14 mm and 18 mm diameter steel bars changing with temperature after high-temperature cooling. Under air cooling, when the heating temperature is below 500 °C, the steel bar surface is dark red (Figure 5a,c). When the heating temperature ranges from 500 °C to 600 °C, the steel bar surface becomes reddish with metallic luster. When the heating temperature is 700 °C–800 °C, the steel surface appears gray black, and the 18 mm diameter steel bar surface darkens. When the test temperature reaches 900 °C–1000 °C, the surface of the steel bars is a deep dark color, and a serious oxide layer forms. The tensile fracture sections of 14 mm and 18 mm diameter steel bars under air cooling both show significant necking and fractures in the shape of a silver cup cone (Figure 5b,d).

The apparent changes of the 14 mm and 18 mm diameter steel bar under furnace cooling are as follows: when the heating temperature does not exceed 500 °C, the steel bar surface appears dark red with metallic luster; when the heating temperature is 500 °C–600 °C, the steel surface is light gray; when the heating temperature is 700 °C–800 °C, the steel surface appears rust red; when the heating temperature is 900 °C–1000 °C, the surface of the steel bar is significantly carbonized, showing a deep dark color, the oxide layer peels off, and the change in the 18 mm diameter steel bar is more significant (Figure 6a,c). Under furnace cooling, the 14 mm and 18 mm diameter steel bar sections contain a silver fracture in the shape of a cup cone with significant necking (Figure 6b,d). When the heating temperature is below 400 °C, the steel surface appears dark gray. When the heating temperature is between 500 °C and 600 °C, the color of the steel bar surface becomes lighter and silver gray. When the heating temperature exceeds 700 °C, the steel surface appears carbonized and the oxide layer fall off.

When the heating temperature is below 400 °C, the 14 mm steel bars after water cooling still have a metallic luster (Figure 7a,c). When the heating temperature is 500 °C–600 °C, the steel bar surface is rust red. When the heating temperature is 700 °C–800 °C, the surface of the steel bar is dark, and shelling occurs. The surface of the 18 mm diameter steel bars is seriously corroded after water cooling. When the heating temperature is 800 °C, the surface of the steel bars is significantly carbonized, and the oxide layer flakes off. The apparent characteristic changes of 14 mm and 18 mm diameter steel bars after water cooling and tensile fracturing are as follows: when the heating temperature does not exceed 400 °C, the surface of the steel bars is reddish, the fracture is still cup-shaped, and necking is significant (Figure 7b,d). When the heating temperature is between 500 °C and 600 °C, the surface of the steel bars appears gray and the fracture is silver gray. When the heating temperature exceeds 700 °C, the oxide layer on the surface of the steel bars flakes off, no necking phenomenon is visible in the fracture, the section is relatively neat, and the steel bars records a brittle fracture [21].

### 3.2. Stress–Strain Curves

**Figure 8:**
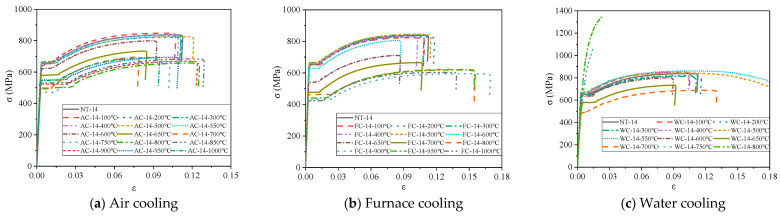
Stress–strain curves of 14 mm diameter steel bars under different cooling conditions after heating.

**Figure 9:**
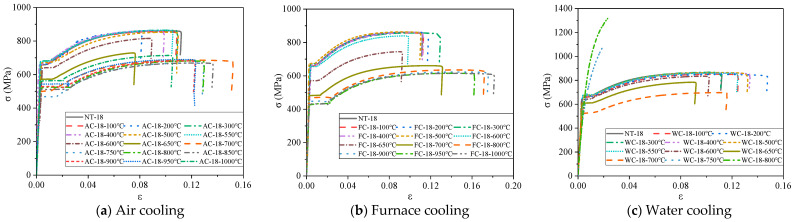
Stress–strain curves of 18 mm diameter steel bars under different cooling conditions after heating.

The stress–strain curves of steel bars under different cooling conditions after high temperature are shown in Figure 8 and Figure 9. The measured stress–strain curves of the steel bars under air cooling are similar to the curves for furnace cooling (Figure 8a,b and Figure 9a,b). When the heating temperature is below 600 °C, the stress–strain curves of the steel bars basically coincide with those at normal temperature, and each of the stress–strain curves is composed of an elastic stage, yield stage, strengthening stage, and necking stage. When the heating temperature exceeds 600 °C, the steel bar stress–strain curves degrade significantly, their conditional yield points and ultimate tensile strength begin to decline, and the yield flow amplitudes increase gradually with increasing heating temperature with decreasing yield steps. When the heating temperature is below 600 °C, the measured stress–strain curves of the steel bars under water cooling are consistent with the normal temperature curve (Figure 8c and Figure 9c). When the heating temperature ranges from 650 °C to 700 °C, the stress–strain curves of steel bars change significantly, and the ultimate strength and yield strength both decrease gradually. When the heating temperature exceeds 700 °C, the yield steps of the steel bar stress–strain curves disappears, and the ultimate strength increases significantly. At 750 °C, the ultimate strength of 14 mm and 18 mm diameter steel bars increases by 29.1% and 25.8%, respectively, compared to the normal temperature. At 800 °C, the ultimate strength of the 14 mm and 18 mm diameter steel bars increases by 61.3% and 52.4%, respectively.

### 3.3. Mechanical Parameters

**Figure 10:**
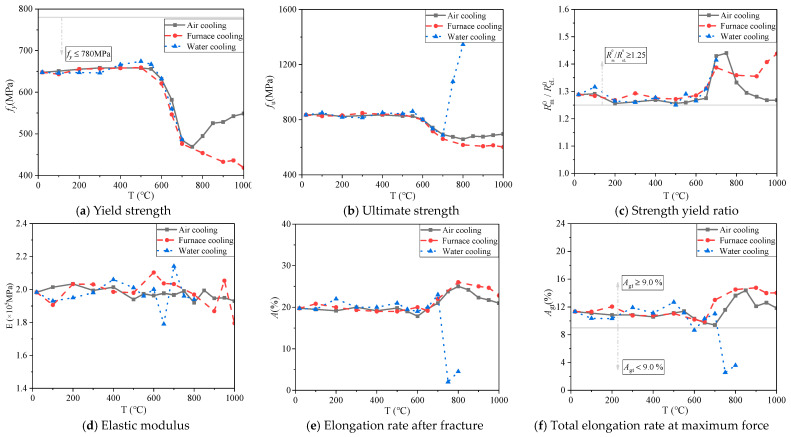
Curves of mechanical indices changing with temperature for 14 mm diameter steel bars under different cooling modes after heating.

**Figure 11:**
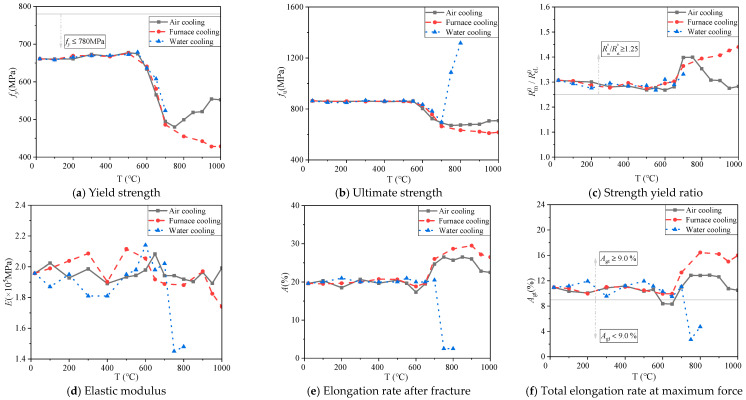
Curves of mechanical indices changing with temperature for 18 mm diameter steel bars under different cooling modes after heating.

Figure 10 and Figure 11 show the change in yield strength, ultimate strength, strength yield ratio, elastic modulus, elongation rate after fracture, and total elongation rate at maximum force of 600 MPa for the 14 mm and 18 mm diameter bars under different cooling conditions. Among them, the calculation expressions of the elongation rate after fracture *A* (%) and the total elongation rate at maximum force *A*_*gt*_ (%) of steel bars refer to GB/T228.1-2010 [26] and GB1499.2-2018 [20], see Equations (1) and (2) below:

(1) $$A=\frac{L_{u}-L_{0}}{L_{0}}\times 100$$

where *L*_0_ is the original standard distance, unit: mm; *L*_*u*_ is the spacing after the break, unit: mm.

(2) $$A_{gt}=\left[\frac{L-L_{b}}{L}+\frac{R_{m}^{0}}{E}\right]\times 100$$

where *L* is the distance after fracture, unit: mm; *L*_*b*_ is the distance between the same standard distance before the test, unit: mm; $R_{m}^{0}$ is the tensile strength of the measured values, unit: MPa; *E* is the elastic modulus, unit: MPa.

(i) Yield strength

When the heating temperature is below 550 °C, the yield strength values of 14 mm and 18 mm diameter steel bars show little difference under the three cooling modes (Figure 10a and Figure 11a). After the temperature exceeds 550 °C, yield strength values begin to decrease, and yield strength values for steel bars under air cooling decrease to the minimum at 750 °C, at which point their values recover. This is because the critical heat treatment temperature of steel generally ranges from 700 to 800 °C. During heating, if the critical temperature is exceeded, the steel will undergo a solid phase transition, resulting in its internal organization as well as structural and performance changes [27]. The yield strength values of the steel bars after furnace cooling decrease continuously with increasing heating temperature and reach the minimum at 1000 °C. The yield strength values of the water-cooled steel bars cannot be calculated because there are no yield steps on the stress–strain curves beyond 700 °C, so the yield strength values are minimized at 700 °C.

(ii) Ultimate strength

When the heating temperature does not exceed 550 °C, different cooling conditions have little impact on the ultimate strength of the 14 mm and 18 mm diameter steel bars (Figure 10b and Figure 11b). When the heating temperature reaches 550 °C, the steel bar ultimate strength decreases gradually under air cooling and furnace cooling, and the decrease becomes greater as the heating temperature continues to rise. However, air cooling, when the heating temperature reaches 800 °C, the steel bar ultimate strength values appear to rise, and at 1000 °C, its value is 695.59 MPa. This phenomenon is similar to the previously mentioned yield strength results. When the heating temperature reaches 700 °C, the ultimate strength values of the water-cooled steel bars increase instead of decrease, and the ultimate strength values of the 14 mm and 18 mm diameter steel bars all reach the maximum value at 800 °C. The ultimate strength values of the 14 mm diameter steel bars increase from 687.94 MPa at 700 °C to the maximum of 1345.89 MPa at 800 °C, and the ultimate strength of the 18 mm diameter steel bars increases from 696.92 MPa at 700 °C to the maximum of 1316.68 MPa at 800 °C, which is due to the fact that water cooling weakens the grain growth of the material, improves its strength and reduces its plasticity [21].

(iii) Strength yield ratio

When the heating temperature is below 650 °C, the strength yield ratio of the 14 mm and 18 mm diameter steel bars varies slightly based on cooling mode (Figure 10c and Figure 11c). When the heating temperature exceeds 650 °C, the variation law of steel bar changes under the three cooling conditions. Under air cooling, the strength yield ratio of the 14 mm and 18 mm diameter steel bars first increases and then decreases. The strength yield ratios of the 14 mm and 18 mm diameter steel bars have maximum values of 1.44 and 1.40, respectively, when the heating temperature reaches 750 °C. Under furnace cooling, the strength yield ratio of the 14 mm diameter steel bars decreases and then increases, while for the 18 mm diameter steel bars, the strength yield ratio increases linearly, reaching a maximum value at 1000 °C, both of which are 1.44. Under water cooling, the strength yield ratio of the 14 mm and 18 mm diameter steel bars begins to increase, with maximums of 1.41 and 1.33, respectively, when the heating temperature reaches 700 °C.

(iv) Elastic modulus

The elastic modulus of the 14 mm diameter steel bars fluctuates to a certain extent with varying heating temperature under different cooling modes (Figure 10d and Figure 11d). It is worth noting that the elastic modulus at 650 °C is abrupt, which requires further study. For the 18 mm diameter steel bars, under air cooling and furnace cooling, elastic modulus varies with temperature change, and furnace cooling of the reinforced elastic modulus range is bigger, change range of 1.74 × 10^5^–2.11 × 10^5^ MPa, but under the condition of immersion cooling steel after heating temperature is 700 °C, sharply lower elastic modulus, 750 °C to 1.45 × 10^5^ MPa, 74.0% of the normal temperature at this time. For the 18 mm diameter steel bars, the elastic modulus fluctuates with the temperature under air cooling and furnace cooling, and the range of elastic modulus values after furnace cooling is larger, with a range of 1.74 × 10^5^–2.11 × 10^5^ MPa. After 700 °C, the elastic modulus of the steel bars after water cooling drops sharply, and at 750 °C, the elastic modulus values of the steel bars decrease to 1.45 × 10^5^ Mpa, which is 74.0% of the normal temperature.

(v) Elongation rate after fracture

When the heating temperature is less than 600 °C, the elongation rates after fracture of the 14 mm and 18 mm diameter steel bars vary little under different cooling conditions (Figure 10e and Figure 11e). When the heating temperature exceeds 600 °C, the elongation rates after fracture of the 14 mm steel bars under air and furnace cooling increase with the temperature overall and reach the maximum at 800 °C, which increase by 26.6% and 31.6%, respectively, compared with normal temperature. The elongation rates after fracture of 18 mm steel bars reach the maximum values of 26.5% and 29.5% at 850 °C and 900 °C, respectively. When the heating temperature reaches 700 °C, the elongation rates after fracturing of the 14 mm and 18mm diameter steel bars after water cooling plummet to the lowest value at 750 °C and 800 °C, which were 10.1% and 12.7% of the normal temperature, respectively. The results of this study are consistent with the conclusions of Sun et al. [21], which is attributed to the fact that when the heating temperature exceeds 700 °C, the steel bars after water cooling show significant brittleness, resulting in a strength reduction [27].

(vi) Total elongation rate at maximum force

The total elongation rates at maximum force for the 14 mm and 18 mm diameter steel bars under different cooling modes vary with heating temperature and are consistent with the elongation rate after fracturing (Figure 10f and Figure 11f). When the heating temperature is less than 600 °C, the change in the total elongation rates at the maximum force is not significant. When the heating temperature reaches 650 °C, the total elongation rate at the maximum force under air cooling and furnace cooling shows an overall increasing trend. However, the total elongation rates at the maximum force of the 14 mm and 18 mm diameter steel bars decrease sharply under water cooling, and both decrease to the minimum values of 2.51% and 2.71% at 750 °C, which are 22.2% and 24.7%, respectively, at room temperature. The result is similar to the variation in elongation rate after fracturing.

### 3.4. Seismic Resistance Analysis

GB50010-2010 [24], and GB/T1499.2-2018 [20] indicate that the total elongation rate at the maximum force of steel bars in seismic structures is greater than or equal to 9.0% (that is *A*_*gt*_ ≥ 9.0%), the measured strength yield ratio $R_{m}^{0}$/$R_{eL}^{0}$ ≥ 1.25, and the ratio of measured yield strength to the theoretical yield strength $R_{eL}^{0}$/*R*_*eL*_ ≤ 1.30. In the CEB-FIP Model Code, it is required that *A*_*gt*_ ≥ 9.0% for “H”-type steel bars in the seismic structure [28]. ASTM A706/A706M-14 requires that the measured strength yield ratio of stressed steel bars $R_{m}^{0}$/$R_{eL}^{0}$ ≥ 1.25 [25]. For 600 MPa steel bars, if $R_{eL}^{0}$/*R*_*eL*_ ≤ 1.30 is required, the measured yield strength values $R_{eL}^{0}$ ≤ 780 MPa is also required. The measured yield strength values $R_{eL}^{0}$ of 14 mm and 18 mm diameter steel bars under three cooling conditions after high-temperature values are all below 780 MPa (Figure 10a and Figure 11a) The measured strength yield ratio $R_{m}^{0}$/$R_{eL}^{0}$ for the 14 mm and 18 mm diameter steel bars under the three cooling conditions after heating simultaneously meets the requirements of GB50010-2010,GB1499.2-2018 and ASTM A706/A706M-14 in terms of the strength yield ratio of seismic steel bars (Figure 10c and Figure 11c). The *A*_*gt*_ values for the 14 mm and 18 mm diameter steel bars after air cooling and furnace cooling both meet the above specification requirements for the total elongation rate at maximum force of seismic steel bars (Figure 10f and Figure 11f). It should be pointed out that the steel bars under the water cooling do not meet the requirements of the above specification for the total elongation rate at maximum force of seismic steel bars when heated above 700 °C.

### 3.5. Calculation Models of Mechanical Parameters

Using the least square method to fit the test results, the calculation model for the mechanical index varying with temperature for 600 MPa seismic steel bars under air cooling after heating can be obtained (Equations (3)–(7)). The *R^2^* (square of correlation coefficient) of the mechanical parameters to the calculation formula for temperature, such as $f_{y}^{T}/f_{y}$, $f_{u}^{T}/f_{u},\;A^{T}/A,\;$ and $A_{gt}^{T}/A_{gt}\;$ are 0.96, 0.89, 0.92, and 0.87, respectively. However, the *R^2^* of $E^{T}/E$ is 0.37, which is relatively low. The test results for steel bars under air cooling heating are in good agreement with the predicted results, indicating that the proposed calculation models can be used to predict the strength and deformation of 600 MPa seismic steel bars under the air-cooling condition after heating.

(3) fyTfy={1    20 °C≤T≤550 °C12.12−40.34(T1000)+47.02(T1000)2−17.97(T1000)3R2=0.96    550 °C<T≤1000 °C

(4) fuTfu={1    20 °C≤T≤550 °C2.51−4.16(T1000)+2.49(T1000)2R2=0.89        550 °C<T≤1000 °C

(5) $$\begin{array}{c} \frac{E^{T}}{E}=0.98+1.07\left(\frac{T}{1000}\right)-8.48{\left(\frac{T}{1000}\right)}^{2}+23.36{\left(\frac{T}{1000}\right)}^{3}-26.44{\left(\frac{T}{1000}\right)}^{4}\\ +10.52{\left(\frac{T}{1000}\right)}^{5}\;\;\;\;\;R^{2}=0.37\;\;\;\;\; \end{array}\;\;20\;{}^{\circ}C<T\leq 1000\;{}^{\circ}C$$

(6) ATA={1    20 °C≤T≤550 °C−4.87+14.98(T1000)−9.01(T1000)2R2=0.92    550 °C<T≤1000 °C

(7) AgtTAgt={1    20 °C≤T≤550 °C−4.89+14.56(T1000)−8.74(T1000)2R2=0.87    550 °C<T≤1000 °C

**Figure 12:**
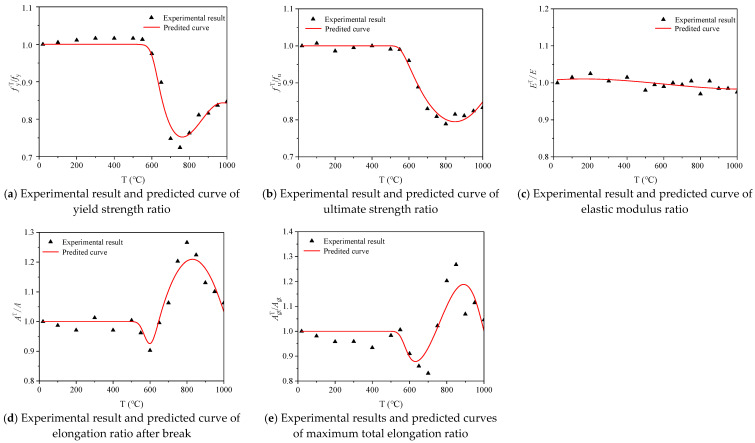
Comparison of experimental results and predicted curves for the mechanical properties of steel bar samples under air cooling.

Figure 12 shows the comparison between the test results and the predicted results of the mechanical properties of 600 MPa seismic steel bars under air cooling after heating. The test results of $f_{y}^{T}/f_{y}$, $f_{u}^{T}/f_{u},\;A^{T}/A$, and $A_{gt}^{T}/A_{gt}\;$ are in good agreement with the predicted results, while the fitting results of $\;E^{T}/E$ are slightly worse. In general, the fitting reliability of the test results and the predicted results for the steel bars under air cooling is high, indicating that the calculation models can be used to predict the mechanical properties of 600 MPa steel bars after heating.

Equations (8)–(12) describe the relationship between reinforcement mechanical parameters and heating temperature, yield strength, ultimate strength, elongation, and the most energetic fits of the total elongation were 0.96, 0.89, 0.93 and 0.90 for furnace cooling, and the *E*^*T*^/*E* fitting degree is 0.57, which is relatively low. Equations (8)–(12) show the relationship between the steel bar mechanical parameters and the heating temperature under furnace cooling. The fitting degrees of $\;f_{y}^{T}/f_{y}$, $f_{u}^{T}/f_{u},\;A^{T}/A$, and $A_{gt}^{T}/A_{gt}$ are 0.96, 0.89, 0.93, and 0.90, respectively, while the fitting degree of $E^{T}/E$ is 0.57, which is relatively low. The results show that the models can be used to predict the mechanical properties of 600 MPa seismic steel bars after cooling.

(8) fyTfy={1    20 °C≤T≤500 °C3.361−5.974(T1000)+3.279(T1000)2R2=0.96    500 °C<T≤1000 °C

(9) fuTfu={1    20 °C≤T≤500 °C2.51−4.16(T1000)+2.49(T1000)2R2=0.89    500 °C<T≤1000 °C

(10) $$\begin{array}{ccc} \frac{E^{T}}{E}=1.002+0.12\left(\frac{T}{1000}\right)-0.289{\left(\frac{T}{1000}\right)}^{2}\;\;\;\;\; & R^{2}=0.57\;\;\;\;\; & 20\;{}^{\circ}C<T\leq 1000\;{}^{\circ}C \end{array}$$

(11) ATA={1    20 °C≤T≤500 °C−4.737+14.426(T1000)−8.355(T1000)2R2=0.93    500 °C<T≤1000 °C

(12) AgtTAgt={1    20 °C≤T≤500 °C−4.381+13.067(T1000)−7.28(T1000)2R2=0.90    500 °C<T≤1000 °C

**Figure 13:**
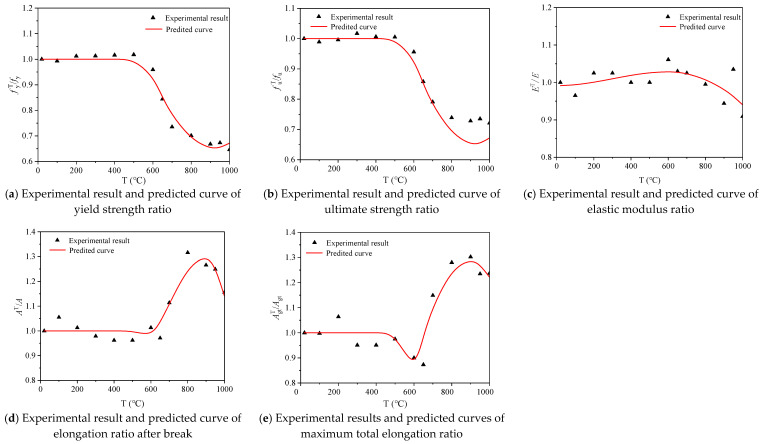
Comparison of experimental results and predicted curves for the mechanical properties of steel bar samples under furnace cooling.

Figure 13 shows the comparison between the test results of the steel bar mechanical properties and the predicted curves under furnace cooling after heating. The test results for mechanical indexes such as $f_{y}^{T}/f_{y}$,$\;f_{u}^{T}/f_{u},\;A^{T}/A,$ and $A_{gt}^{T}/A_{gt}\;$ of the steel bars are in agreement with the predicted curves, while the test results of $E^{T}/E$ have a lower agreement with the predicted curves. Overall, the test results of the steel bars under furnace cooling after heating are in good agreement with the prediction curves, indicating that the calculation models can predict the mechanical properties of 600 MPa steel bars after heating.

Equations (13)–(17) show the relationship between the heating temperature and mechanical indices ($f_{y}^{T}/f_{y}$,$\;f_{u}^{T}/f_{u}$, $E^{T}/E$, $A^{T}/A$, and $A_{gt}^{T}/A_{gt}$) of the steel bars under after water cooling after heating, and they are all piecewise functions with fitting degrees of 0.95, 0.92, 0.71, 0.75, and 0.53, respectively. The relationship between $f_{y}^{T}/f_{y}$, $f_{u}^{T}/f_{u}$, and heating temperature of steel bars has a high degree of agreement, while the agreement between other indexes and heating temperature is relatively low, indicating that the calculation models can reasonably predict the yield strength and ultimate strength of 600 MPa seismic steel bars under water cooling and predict the elastic modulus and elongation rate after fracture. The total elongation rate at maximum force requires further examination.

(13) fyTfy={1    20 °C≤T≤500 °C1.861−1.496(T1000)R2=0.95    500 °C<T≤700 °C

(14) fuTfu={1    20 °C≤T≤550 °C15.65−45.02(T1000)+34.26(T1000)2R2=0.92    550 °C<T≤800 °C

(15) ETE={1    20 °C≤T≤650 °C2.912−2.76(T1000)R2=0.71    650 °C<T≤800 °C

(16) ATA={1    20 °C≤T≤650 °C7.310−9.17(T1000)R2=0.75    650 °C<T≤800 °C

(17) AgtTAgt={1    20 °C≤T≤650 °C4.891−5.77(T1000)R2=0.53    650 °C<T≤800 °C

**Figure 14:**
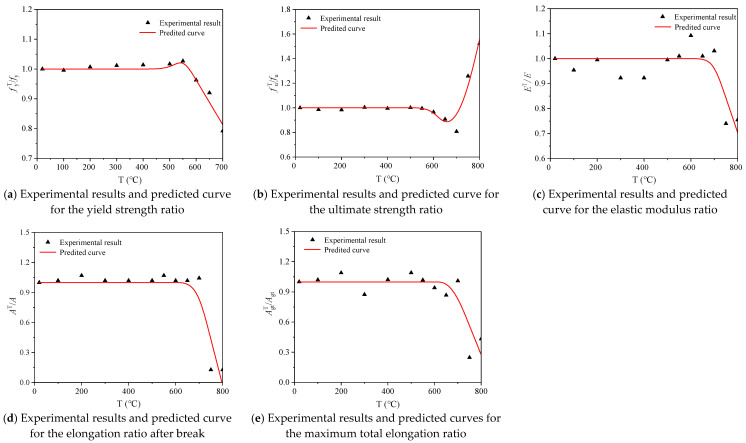
Comparison of experimental results and predicted curves of the mechanical properties of steel bar samples under water cooling.

Figure 14 shows the comparison between the test results and the predicted curves for the mechanical properties of steel bars under water cooling after heating. The test results of $f_{y}^{T}/f_{y}\;$ and $\;f_{u}^{T}/f_{u}$ of steel bars are in good agreement with their predicted curves, while the test results of $E^{T}/E$, $A^{T}/A,\;$ and $\;A_{gt}^{T}/A_{gt}$ are slightly less in agreement with their predicted curves, indicating that the proposed calculation models can predict the strength of water-cooled 600 MPa steel bars, while the prediction of their deformation indices requires further study.

### 3.6. Constitutive Models

The stress–strain relationship model of materials under a monotone load, also known as the constitutive model of materials, is a mathematical expression describing the mechanical properties of materials. An appropriate constitutive model can obtain an accurate structural response and mechanical properties of materials as well as provide theoretical guidance for engineers in terms of structural design and numerical simulation [29]. According to the stress–strain characteristics of the 600 MPa seismic steel bars under different cooling modes after heating, except for water-cooled steel bars above 700 °C, the stress–strain curves of the steel bars under other cooling conditions are still in the elastic stage, yield stage, and strengthening stage, and the yield step is significant. Therefore, the two-fold line and three-fold line constitutive models are considered to describe the stress–strain relationship of steel bars after high-temperature cooling (see Equations (18)–(22)). In addition, all steel bars show significant nonlinear strengthening behavior under the various cooling methods; hence, the Ramberg–Osgood model is used to describe the stress–strain relationship of steel bars [29] (see Equation (23)).

Two-fold line constitutive model:

(18) $$\begin{array}{cc} \sigma =E^{T}\cdot \varepsilon \;\;\;\;\;\;\;\;\;\; & \left(\varepsilon \leq \varepsilon_{y}^{T}\right) \end{array}$$

(19) $$\begin{array}{cc} \sigma =\sigma_{0}^{T}\;+E_{t}^{T}\left(\varepsilon -\varepsilon_{y}\right)\;\;\;\;\;\;\;\;\;\; & \left(\varepsilon_{y}^{T}<\varepsilon \leq \varepsilon_{u}^{T}\right) \end{array}$$

Three-fold line constitutive model:

(20) $$\begin{array}{cc} \sigma =E^{T}\cdot \varepsilon \;\;\;\;\;\;\;\;\;\; & \left(\varepsilon \leq \varepsilon_{y}^{T}\right) \end{array}$$

(21) $$\begin{array}{cc} \sigma =\sigma_{0}^{T}\;\;\;\;\;\;\;\;\;\; & \left(\varepsilon_{y}^{T}<\varepsilon \leq \varepsilon_{sh}^{T}\right) \end{array}$$

(22) $$\begin{array}{cc} \sigma =\sigma_{0}^{T}+E_{t}^{T}\left(\varepsilon -\varepsilon_{sh}^{T}\right)\;\;\;\;\;\;\;\;\;\; & \left(\varepsilon_{sh}^{T}<\varepsilon \leq \varepsilon_{u}^{T}\right) \end{array}$$

Ramberg–Osgood model:

(23) $$\begin{array}{cc} \varepsilon =\frac{\sigma }{E^{T}}+\alpha \times \frac{\sigma_{0}^{T}}{E^{T}}{\left(\frac{\sigma }{\sigma_{0}^{T}}\right)}^{n}\;\;\;\;\;\; & \left(\varepsilon \leq \varepsilon_{u}^{T}\right) \end{array}$$

#### 3.6.1. Two-Fold Line Constitutive Model

When the two-fold line constitutive simulation of the 600 Mpa seismic steel bars under air, furnace, and water cooling is carried out, determining three basic parameters is necessary, namely, the elastic modulus E of the material, the measured yield strength *σ*_0_, and the reinforced stiffness *E*_*t*_. Three basic parameters, i.e., elastic modulus *E*, measured yield strength *σ*_0_ and strengthened stiffness *E*_*t*_ need to be determined for the constitutive simulation of 600 MPa seismic steel bars under the three cooling conditions after high temperature. *σ*_0_ can be obtained according to material test, and *E* and *E*_*t*_ can be obtained by fitting the results of the straight-pull test (Table 1, Table 2 and Table 3). For specific values, please refer to Table 1, Table 2 and Table 3. The ratio of *E*_*t*_ to *E* for seismic steel bars in different cooling modes after heating is 0.01, indicating that different heating temperature and cooling conditions have little impact on the relationship between *E* and *E*_*t*_ for steel bars. Therefore, it is suggested that *E*_*t*_ = 0.01*E* in the two-fold constitutive model of 600 MPa seismic steel bars.

#### 3.6.2. Three-Fold Line Constitutive Model

In the process of simulating the three-fold line constitutive model of 600 MPa seismic steel bars under the air cooling, furnace cooling, and water cooling conditions after high temperature, it is necessary to determine basic parameters, such as the elastic modulus *E* of the material, the measured yield strength σ_0_, the reinforced stiffness *E*_*t*_, and the end strain *ε*_*sh*_ of the yield stage (Table 4, Table 5 and Table 6). Please refer to Table 4, Table 5 and Table 6 for details. The ratio of *E*_*t*_ to elastic modulus *E* of steel bars under different cooling modes after high temperature is 0.01. The results are consistent with the relationship between *E*_*t*_ and elastic modulus *E* given by Guan et al. [23]. Therefore, it is suggested that the parameter *E*_*t*_ of the three-fold line constitutive model for 600 MPa seismic steel bars after heating be 0.01*E*.

#### 3.6.3. Ramberg–Osgood model

In Ramberg–Osgood constitutive simulation, it is necessary to determine the elastic modulus *E*, yield strength *σ*_0_, as well as the unknown parameters α and n of steel bars. For the case that there is no yield step on the stress–strain curve of the material, when determining its elastic modulus *E*, the value can be determined according to the slope at the point where the linearity is significant in the elastic stage of the stress–strain curve. In this case, *σ*_0_ is taken as the conditional yield strength *σ*_0.2_ of the steel bars. As for the unknown parameters α and n, they can be obtained using by least squares fitting. Please refer to Table 7, Table 8 and Table 9 for details. α and n in the nonlinear equation of steel specimens under the action of different temperatures do not change significantly; however, by observing three different cooling methods, it is found that under after water cooling at 750 °C and 800 °C, the values of α and n are smaller than those for air cooling and furnace cooling.

#### 3.6.4. Comparative Analysis of Constitutive Model

**Figure 15:**
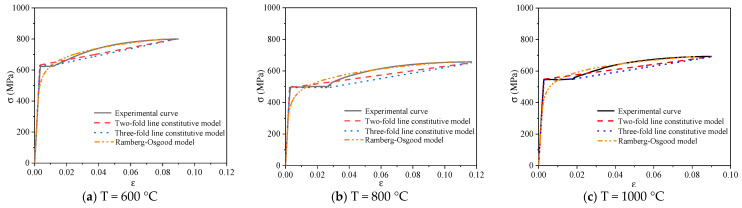
Comparison of the constitutive models of typical steel bars under air cooling.

**Figure 16:**
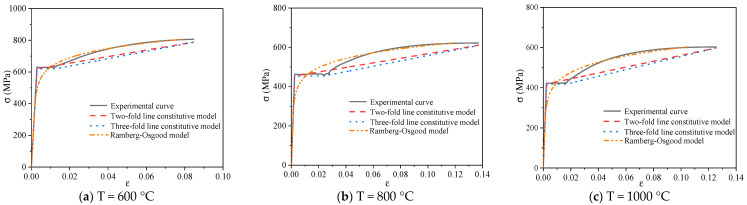
Comparison of the constitutive models of typical steel bars under furnace cooling.

**Figure 17:**
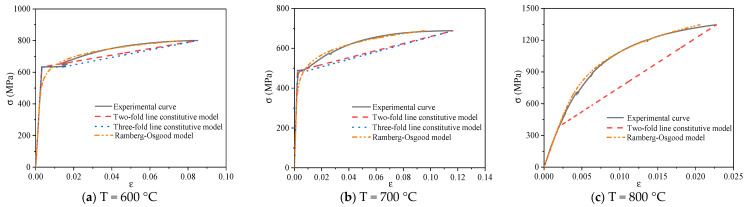
Comparison of the constitutive models of typical steel bars under water cooling.

Figure 15, Figure 16 and Figure 17 show the typical constitutive model comparison for 600 MPa steel bars under different cooling modes after heating. The two-fold line model, three-fold line model, and Ramberg–Osgood model can simulate the stress–strain curves of steel bars under different cooling modes after heating. Among them, the two-fold line constitutive equation has a simple form, which can be used for simplifying the simulation of the constitutive relationship of 600 MPa steel bars after heating. The three-fold line model can simulate the case of significant yield step in the stress–strain curves of 600 MPa steel bars. The Ramberg–Osgood model is a nonlinear curve model, which can simulate the stress–strain relationship of 600 MPa steel bars after heating. The Ramberg–Osgood model is best in the strengthening stage.

## 4. Conclusions

In this contribution, high-temperature tests and tensile tests were carried out on 600 MPa seismic steel bars under three cooling modes after heating. The change in apparent characteristics of steel bars was observed, the stress–strain curves were obtained, the change of mechanical indexes with heating temperature was analyzed, and suitable constitutive models were proposed for 600 MPa seismic steel bars through different cooling rate. The main conclusions are as follows:

(1) The various cooling methods have significant effects on the apparent characteristics of steel bars. When heating temperature is lower than 600 °C, the apparent characteristics of reinforcement under the three cooling conditions shows little variance. When the heating temperature is greater than 600 °C, the surface of the steel bars under air cooling and furnace cooling is reddish brown, and the surface of the steel bars under water cooling is seriously corroded. When the heating temperature reaches 700 °C, the surface of steel bars is seriously carbonized, and the oxide layer falls off.

(2) After heating, the yield strength of steel bars cooled by air, furnace, and water decreases with increasing heating temperature, but the yield strength of steel bars cooled by air increases after 750 °C. The ultimate strength decreases with increasing heating temperature, while the ultimate strength of steel bars cooled by water increases after 750 °C. The elastic modulus of steel bars is almost not affected by cooling method. When the heating temperature is less than 650 °C, the elongation rate after fracture and the total elongation rate at the maximum force do not change significantly. After 650 °C, the elongation rate after fracture and the total elongation rate at the maximum force of steel bars under air and furnace cooling show an overall upward trend, while the elongation rate after fracture and the total elongation rate at the maximum force of steel bars under water cooling drop sharply.

(3) When the heating temperature is high, the stress–strain curves of steel bars degrade, and the strengthening stage is shortened. After 700 °C, there is no yield step in the stress–strain curves of the water-cooled steel bars, and the ultimate strength of the steel bars increases significantly, because water cooling weakens the grain growth of the steel, increases its strength, reduces its plasticity, and causes brittle fracturing of the steel bars.

(4) The relationship between each mechanical index and heating temperature is described. The strength results under the three cooling methods are in good agreement with the predicted results, while the deformation test results for water cooling are quite different from the predicted results.

(5) The constitutive models for 600 MPa seismic steel bars after high-temperature cooling (air cooling, furnace cooling and water cooling) were proposed. The two-fold line, three-fold line, and Ramberg–Osgood models for the new 600 MPa seismic steel bars are presented. The two-fold line model is suitable for the simplified simulation of the stress–strain relationship of steel bars after heating. The three-fold line model can better describe the stress–strain relationship of steel bars with a significant yield step after heating. The Ramberg–Osgood model is the best for simulating the strengthening stage of the stress–strain curve of steel bars after heating.

(6) The mechanical index calculation method of 600 MPa seismic steel bars after high-temperature cooling (air cooling, furnace cooling and water cooling) is established.

## Figures and Tables

**Table 1:** The parameters of two-fold constitutive model of steel bars under air cooling.

Heating Temperature	$\sigma =E^{T}\cdot \varepsilon \;\;\;\;\;\left(\varepsilon \leq \varepsilon_{y}^{T}\right)\;\;\;$		$\sigma =\sigma_{0}^{T}+E_{t}^{T}\left(\varepsilon -\varepsilon_{y}\right)\;\;\;\;\;\;(\varepsilon_{y}^{T}<\varepsilon \leq \varepsilon_{u}^{T})$		
	Elastic Modulus *E*^*T*^ × 10^5^ (MPa)	Value Range	Yield Strength $\sigma_{0}^{T}$ (MPa)	Strengthen Stiffness $E_{t}^{T}$ × 10^5^ (MPa)	Value Range
100 °C	2.01	ε ≤ 0.0032	650.92	0.021	0.0032 < ε ≤ 0.0917
200 °C	2.03	ε ≤ 0.0032	655.36	0.018	0.0032 < ε ≤ 0.0986
300 °C	1.99	ε ≤ 0.0033	658.49	0.019	0.0033 < ε ≤ 0.0962
400 °C	2.01	ε ≤ 0.0033	658.26	0.018	0.0033 < ε ≤ 0.1030
500 °C	1.94	ε ≤ 0.0034	658.19	0.016	0.0034 < ε ≤ 0.1078
550 °C	1.97	ε ≤ 0.0033	656.04	0.018	0.0033 < ε ≤ 0.0991
600 °C	1.96	ε ≤ 0.0032	631.72	0.020	0.0032 < ε ≤ 0.0888
650 °C	1.98	ε ≤ 0.0029	581.62	0.021	0.0029 < ε ≤ 0.0802
700 °C	1.97	ε ≤ 0.0025	484.73	0.024	0.0025 < ε ≤ 0.0889
750 °C	1.99	ε ≤ 0.0024	468.92	0.021	0.0024 < ε ≤ 0.1030
800 °C	1.92	ε ≤ 0.0026	494.58	0.014	0.0026 < ε ≤ 0.1216
850 °C	1.99	ε ≤ 0.0026	525.57	0.013	0.0026 < ε ≤ 0.1201
900 °C	1.95	ε ≤ 0.0027	528.40	0.013	0.0027 < ε ≤ 0.1183
950 °C	1.95	ε ≤ 0.0028	542.43	0.014	0.0028 < ε ≤ 0.1090
1000 °C	1.93	ε ≤ 0.0028	548.72	0.016	0.0028 < ε ≤ 0.0925

**Table 2:** The parameters of two-fold constitutive model of steel bars under furnace cooling.

Heating Temperature	$\sigma =E^{T}\cdot \varepsilon \;\;\;\;\;\left(\varepsilon \leq \varepsilon_{y}^{T}\right)\;\;\;$		$\sigma =\sigma_{0}^{T}+E_{t}^{T}\left(\varepsilon -\varepsilon_{y}\right)\;\;\;\;\;\;(\varepsilon_{y}^{T}<\varepsilon \leq \varepsilon_{u}^{T})$		
	Elastic Modulus *E*^*T*^ × 10^5^ (MPa)	Value Range	Yield Strength $\sigma_{0}^{T}$ (MPa)	Strengthen Stiffness $E_{t}^{T}$ × 10^5^ (MPa)	Value Range
100 °C	1.91	ε ≤ 0.0034	643.31	0.020	0.0034 < ε ≤ 0.0923
200 °C	2.03	ε ≤ 0.0032	655.55	0.019	0.0032 < ε ≤ 0.0955
300 °C	2.03	ε ≤ 0.0032	656.52	0.020	0.0032 < ε ≤ 0.0992
400 °C	1.98	ε ≤ 0.0033	658.03	0.019	0.0033 < ε ≤ 0.0971
500 °C	1.98	ε ≤ 0.0033	659.32	0.017	0.0033 < ε ≤ 0.1071
600 °C	2.10	ε ≤ 0.0030	621.05	0.021	0.0030 < ε ≤ 0.0891
650 °C	2.04	ε ≤ 0.0027	546.58	0.021	0.0027 < ε ≤ 0.0854
700 °C	2.03	ε ≤ 0.0023	476.05	0.017	0.0023 < ε ≤ 0.1109
800 °C	1.97	ε ≤ 0.0023	454.01	0.012	0.0023 < ε ≤ 0.1424
900 °C	1.87	ε ≤ 0.0023	432.78	0.013	0.0023 < ε ≤ 0.1330
950 °C	2.05	ε ≤ 0.0021	436.15	0.014	0.0021 < ε ≤ 0.1327
1000 °C	1.80	ε ≤ 0.0023	418.59	0.015	0.0023 < ε ≤ 0.1278

**Table 3:** The parameters of two-fold constitutive model of steel bars under water cooling.

Heating Temperature	$\sigma =E^{T}\cdot \varepsilon \;\;\;\;\;\left(\varepsilon \leq \varepsilon_{y}^{T}\right)\;\;\;$		$\sigma =\sigma_{0}^{T}+E_{t}^{T}\left(\varepsilon -\varepsilon_{y}\right)\;\;\;\;\;\;(\varepsilon_{y}^{T}<\varepsilon \leq \varepsilon_{u}^{T})$		
	Elastic Modulus *E*^*T*^ × 10^5^ (MPa)	Value Range	Yield Strength $\sigma_{0}^{T}$ (MPa)	Strengthen Stiffness $E_{t}^{T}$ × 10^5^ (MPa)	Value Range
100 °C	1.93	ε ≤ 0.0033	645.70	0.022	0.0033 < ε ≤ 0.0947
200 °C	1.95	ε ≤ 0.0033	647.20	0.018	0.0033 < ε ≤ 0.0991
300 °C	1.98	ε ≤ 0.0033	646.54	0.018	0.0033 < ε ≤ 0.0983
400 °C	2.06	ε ≤ 0.0032	666.28	0.021	0.0032 < ε ≤ 0.0929
500 °C	2.01	ε ≤ 0.0034	674.22	0.018	0.0034 < ε ≤ 0.0963
550 °C	1.96	ε ≤ 0.0034	667.18	0.019	0.0034 < ε ≤ 0.1065
600 °C	2.00	ε ≤ 0.0032	632.61	0.020	0.0032 < ε ≤ 0.0850
650 °C	1.79	ε ≤ 0.0031	560.16	0.020	0.0031 < ε ≤ 0.0887
700 °C	2.14	ε ≤ 0.0023	486.39	0.018	0.0023 < ε ≤ 0.1163
750 °C	1.84 *	ε ≤ 0.0020	349.77	0.555	0.0020 < ε ≤ 0.0151
800 °C	2.00 *	ε ≤ 0.0020	384.42	0.464	0.0020 < ε ≤ 0.0227

Note: * denotes the elastic modulus values of the steel bars are determined by tangent method under the water-cooling condition, when the heating temperature is 750 °C and 800 °C.

**Table 4:** The parameters of the three-fold constitutive model of steel bar samples under air cooling.

Heating Temperature	$\sigma =E^{T}\cdot \varepsilon \;\;\;\;\;\left(\varepsilon \leq \varepsilon_{y}^{T}\right)$		$\sigma =\sigma_{0}^{T}\;\;\;\;(\varepsilon_{y}^{T}<\varepsilon \leq \varepsilon_{sh}^{T})$		$\sigma =\sigma_{0}^{T}+E_{t}^{T}\left(\varepsilon -\varepsilon_{sh}^{T}\right)$ $\left(\varepsilon_{sh}^{T}<\varepsilon \leq \varepsilon_{u}^{T}\right)$	
	Elastic Modulus *E*^*T*^ × 10^5^ (MPa)	Value Range	Yield Strength $\sigma_{0}^{T}$ (MPa)	Value Range	Strengthen Stiffness $E_{t}^{T}$ × 10^5^ (MPa)	Value Range
100 °C	2.01	ε ≤ 0.0032	650.92	0.0032 < ε ≤ 0.0083	0.023	0.0083 < ε ≤ 0.0917
200 °C	2.03	ε ≤ 0.0032	655.36	0.0032 < ε ≤ 0.0135	0.020	0.0135 < ε ≤ 0.0986
300 °C	1.99	ε ≤ 0.0033	658.49	0.0033 < ε ≤ 0.0141	0.021	0.0141 < ε ≤ 0.0962
400 °C	2.01	ε ≤ 0.0033	658.26	0.0033 < ε ≤ 0.0130	0.020	0.0130 < ε ≤ 0.1030
500 °C	1.94	ε ≤ 0.0034	658.19	0.0034 < ε ≤ 0.0158	0.018	0.0158< ε ≤ 0.1078
550 °C	1.97	ε ≤ 0.0033	656.04	0.0033 < ε ≤ 0.0160	0.020	0.0160 < ε ≤ 0.9991
600 °C	1.96	ε ≤ 0.0032	631.72	0.0032 < ε ≤ 0.0122	0.022	0.0122 < ε ≤ 0.0888
650 °C	1.98	ε ≤ 0.0029	581.62	0.0029 < ε ≤ 0.0170	0.025	0.0170 < ε ≤ 0.0802
700 °C	1.97	ε ≤ 0.0025	484.73	0.0025 < ε ≤ 0.0096	0.026	0.0096 < ε ≤ 0.0889
750 °C	1.99	ε ≤ 0.0024	468.92	0.0024 < ε ≤ 0.0137	0.023	0.0137 < ε ≤ 0.1030
800 °C	1.92	ε ≤ 0.0026	494.58	0.0026 < ε ≤ 0.0269	0.017	0.0269 < ε ≤ 0.1216
850 °C	1.99	ε ≤ 0.0026	525.57	0.0026 < ε ≤ 0.0257	0.016	0.0257 < ε ≤ 0.1201
900 °C	1.95	ε ≤ 0.0027	528.40	0.0027 < ε ≤ 0.0254	0.016	0.0254 < ε ≤ 0.1183
950 °C	1.95	ε ≤ 0.0028	542.43	0.0028 < ε ≤ 0.0233	0.017	0.0233 < ε ≤ 0.1090
1000 °C	1.93	ε ≤ 0.0028	548.72	0.0028 < ε ≤ 0.0178	0.020	0.0178 < ε ≤ 0.0925

**Table 5:** The parameters of the three-fold constitutive model of steel bar samples under furnace cooling.

Heating Temperature	$\sigma =E^{T}\cdot \varepsilon \;\;\;\;\;\left(\varepsilon \leq \varepsilon_{y}^{T}\right)$		$\sigma =\sigma_{0}^{T}\;\;\;\;(\varepsilon_{y}^{T}<\varepsilon \leq \varepsilon_{sh}^{T})$		$\sigma =\sigma_{0}^{T}+E_{t}^{T}\left(\varepsilon -\varepsilon_{sh}^{T}\right)$ $\left(\varepsilon_{sh}^{T}<\varepsilon \leq \varepsilon_{u}^{T}\right)$	
	Elastic Modulus *E*^*T*^ × 10^5^ (MPa)	Value Range	Yield Strength $\sigma_{0}^{T}$ (MPa)	Value Range	Strengthen Stiffness $E_{t}^{T}$ × 10^5^ (MPa)	Value Range
100 °C	1.91	ε ≤ 0.0034	643.31	0.0034 < ε ≤ 0.0138	0.023	0.0138 < ε ≤ 0.0923
200 °C	2.03	ε ≤ 0.0032	655.55	0.0032 < ε ≤ 0.0148	0.022	0.0148 < ε ≤ 0.0955
300 °C	2.03	ε ≤ 0.0032	656.52	0.0032 < ε ≤ 0.0128	0.022	0.0128 < ε ≤ 0.0992
400 °C	1.98	ε ≤ 0.0033	658.03	0.0033 < ε ≤ 0.0149	0.022	0.0149 < ε ≤ 0.0971
500 °C	1.98	ε ≤ 0.0033	659.32	0.0033 < ε ≤ 0.0113	0.019	0.0113 < ε ≤ 0.1071
600 °C	2.10	ε ≤ 0.0030	621.05	0.0030 < ε ≤ 0.0130	0.023	0.0130 < ε ≤ 0.0891
650 °C	2.04	ε ≤ 0.0027	546.58	0.0027 < ε ≤ 0.0124	0.023	0.0124 < ε ≤ 0.0854
700 °C	2.03	ε ≤ 0.0023	476.05	0.0023 < ε ≤ 0.0131	0.019	0.0131 < ε ≤ 0.1109
800 °C	1.97	ε ≤ 0.0023	454.01	0.0023 < ε ≤ 0.0238	0.014	0.0238 < ε ≤ 0.1424
900 °C	1.87	ε ≤ 0.0023	432.78	0.0023 < ε ≤ 0.0238	0.016	0.0238 < ε ≤ 0.1330
950 °C	2.05	ε ≤ 0.0021	436.15	0.0021 < ε ≤ 0.0203	0.016	0.0203 < ε ≤ 0.1327
1000 °C	1.80	ε ≤ 0.0023	418.59	0.0023 < ε ≤ 0.0168	0.016	0.0168 < ε ≤ 0.1278

**Table 6:** The parameters of the three-fold constitutive model of steel bar samples under water cooling.

Heating Temperature	$\sigma =E^{T}\cdot \varepsilon \;\;\;\;\;\left(\varepsilon \leq \varepsilon_{y}^{T}\right)$		$\sigma =\sigma_{0}^{T}\;\;\;\;(\varepsilon_{y}^{T}<\varepsilon \leq \varepsilon_{sh}^{T})$		$\sigma =\sigma_{0}^{T}+E_{t}^{T}\left(\varepsilon -\varepsilon_{sh}^{T}\right)$ $\left(\varepsilon_{sh}^{T}<\varepsilon \leq \varepsilon_{u}^{T}\right)$	
	Elastic Modulus *E*^*T*^ × 10^5^ (MPa)	Value Range	Yield Strength $\sigma_{0}^{T}$ (MPa)	Value Range	Strengthen Stiffness $E_{t}^{T}$ × 10^5^ (MPa)	Value Range
100 °C	1.93	ε ≤ 0.0033	645.70	0.0033 < ε ≤ 0.0092	0.024	0.0092 < ε ≤ 0.0947
200 °C	1.95	ε ≤ 0.0033	647.20	0.0033 < ε ≤ 0.0146	0.020	0.0146 < ε ≤ 0.0991
300 °C	1.98	ε ≤ 0.0033	646.54	0.0033 < ε ≤ 0.0142	0.020	0.0142 < ε ≤ 0.0983
400 °C	2.06	ε ≤ 0.0032	666.28	0.0032 < ε ≤ 0.0104	0.022	0.0104 < ε ≤ 0.0929
500 °C	2.01	ε ≤ 0.0034	674.22	0.0034 < ε ≤ 0.0100	0.020	0.0100 < ε ≤ 0.0963
550 °C	1.96	ε ≤ 0.0034	667.18	0.0034 < ε ≤ 0.0129	0.021	0.0129 < ε ≤ 0.1065
600 °C	2.00	ε ≤ 0.0032	632.61	0.0032 < ε ≤ 0.0141	0.024	0.0141 < ε ≤ 0.0850
650 °C	1.79	ε ≤ 0.0031	560.16	0.0031 < ε ≤ 0.0178	0.024	0.0178 < ε ≤ 0.0887
700 °C	2.14	ε ≤ 0.0023	486.39	0.0023 < ε ≤ 0.0092	0.019	0.0092 < ε ≤ 0.1163
750 °C	1.84	-	-	-	-	-
800 °C	2.00	-	-	-	-	-

Note: When the heating temperature is 750 °C and 800 °C, the stress–strain curves of the steel bars do not have yield step, so the three-fold line simulation cannot be carried out.

**Table 7:** The parameters of the Ramberg–Osgood model of steel bars under natural-cooling condition.

Heating Temperature	$\varepsilon =\frac{\sigma }{E^{T}}+\alpha \times \frac{\sigma_{0}^{T}}{E^{T}}{\left(\frac{\sigma }{\sigma_{0}^{T}}\right)}^{n}\;\;\;\;\;\;\left(\varepsilon \leq \varepsilon_{u}^{T}\right)$			
	Elastic Modulus *E*^*T*^ × 10^5^ (MPa)	Yield Strength $\sigma_{0}^{T}$ (MPa)	*α*	*n*
100 °C	2.01	650.92	1.56	10.34
200 °C	2.03	655.36	2.03	11.47
300 °C	1.99	658.49	1.58	11.51
400 °C	2.01	658.26	1.89	10.77
500 °C	1.94	658.19	1.96	11.30
550 °C	1.97	656.04	2.08	10.80
600 °C	1.96	631.72	2.35	9.82
650 °C	1.98	581.62	2.93	9.34
700 °C	1.97	484.73	2.17	7.06
750 °C	1.99	468.92	2.92	7.16
800 °C	1.92	494.58	3.85	8.15
850 °C	1.99	525.57	3.29	9.43
900 °C	1.95	528.40	3.96	9.58
950 °C	1.95	542.43	2.87	9.91
1000 °C	1.93	548.72	2.84	9.75

**Table 8:** The parameters of the Ramberg–Osgood model of steel bars under furnace-cooling condition.

Heating Temperature	$\varepsilon =\frac{\sigma }{E^{T}}+\alpha \times \frac{\sigma_{0}^{T}}{E^{T}}{\left(\frac{\sigma }{\sigma_{0}^{T}}\right)}^{n}\;\;\;\;\;\;\left(\varepsilon \leq \varepsilon_{u}^{T}\right)$			
	Elastic Modulus *E*^*T*^ × 10^5^ (MPa)	Yield Strength $\sigma_{0}^{T}$ (MPa)	*α*	*n*
100 °C	1.91	643.31	1.40	11.31
200 °C	2.03	655.55	1.97	10.87
300 °C	2.03	656.52	1.74	10.56
400 °C	1.98	658.03	1.81	11.03
500 °C	1.98	659.32	1.57	11.07
600 °C	2.10	621.05	2.05	9.65
650 °C	2.04	546.58	2.79	8.74
700 °C	2.03	476.05	2.76	7.72
800 °C	1.97	454.01	3.57	8.37
900 °C	1.87	432.78	3.90	8.08
950 °C	2.05	436.15	3.32	7.91
1000 °C	1.80	418.59	2.84	7.46

**Table 9:** The parameters of the Ramberg–Osgood model of steel bars under water-cooling condition.

Heating Temperature	$\varepsilon =\frac{\sigma }{E^{T}}+\alpha \times \frac{\sigma_{0}^{T}}{E^{T}}{\left(\frac{\sigma }{\sigma_{0}^{T}}\right)}^{n}\;\;\;\;\;\;\left(\varepsilon \leq \varepsilon_{u}^{T}\right)$			
	Elastic Modulus *E*^*T*^ × 10^5^ (MPa)	Yield Strength $\sigma_{0}^{T}$ (MPa)	*α*	*n*
100 °C	1.93	645.70	1.30	10.36
200 °C	1.95	647.20	1.85	10.81
300 °C	1.98	646.54	1.72	11.52
400 °C	2.06	666.28	1.60	10.94
500 °C	2.01	674.22	1.39	12.39
550 °C	1.96	667.18	1.31	11.39
600 °C	2.00	632.61	1.82	10.82
650 °C	1.79	560.16	1.88	9.58
700 °C	2.14	486.39	2.08	8.56
750 °C	1.96	645.70	0.08	3.65
800 °C	1.94	647.20	0.01	5.25

## Data Availability

The data presented in this study are available on request the corresponding author.
